# Surgical Management of Penile Calciphylaxis Without Penectomy

**DOI:** 10.7759/cureus.69677

**Published:** 2024-09-18

**Authors:** Nader Shah, Jay Xiong, Haider Shah

**Affiliations:** 1 Urology, Touro University California, Stockton, USA; 2 Internal Medicine, St. Joseph’s Medical Center, Stockton, USA

**Keywords:** urology, uremic calciphylaxis, calciphylaxis, penile gangrene, partial penectomy, treatment of calciphylaxis, penile calciphylaxis

## Abstract

Calciphylaxis, a rare and life-threatening condition, involves the calcification and occlusion of microvasculature, leading to tissue ischemia and necrosis. The pathophysiology of calciphylaxis remains complex, but it is often associated with derangements in calcium and phosphate metabolism, ultimately resulting in the deposition of calcium within small blood vessels. This process leads to compromised blood flow, tissue hypoxia, and subsequent skin necrosis and ulceration, often with catastrophic consequences. While calciphylaxis typically occurs in individuals with end-stage renal disease (uremic calciphylaxis), it can also afflict those without renal impairment (non-uremic calciphylaxis). Several risk factors predispose individuals to this condition, including diabetes mellitus, hyperparathyroidism, malignancies, warfarin-based anticoagulation, alcoholic liver disease, and autoimmune disorders. Understanding the etiology, risk factors, and clinical manifestations of calciphylaxis is critical for timely diagnosis and management to mitigate its devastating effects. Management includes sepsis control, wound debridement, and analgesic support.

We report a case of penile calciphylaxis in a 58-year old male with a past medical history significant for end stage renal disease on hemodialysis, diabetes mellitus, and hypertension. The patient presented with a painful lesion on the glans penis which rapidly progressed to necrosis and gangrene with wet features. The patient refused partial penectomy and wanted conservative management with local wound debridement and antibiotics.

## Introduction

Calcific uremic arteriolopathy, also known as systemic calciphylaxis, is an uncommon and severe disease characterized by skin necrosis and ulceration. Currently, the exact pathophysiology of calciphylaxis is incompletely understood, but it is often associated with dysregulation of calcium and phosphate metabolism, ultimately resulting in medial calcification, thrombosis, and obliteration of small arterioles within adipose tissue and dermal layers [1]. This process leads to compromised blood flow, tissue hypoxia, and subsequent skin necrosis and ulceration. This condition is highly fatal and reported to have a 1-year mortality rate greater than 50 percent, most frequently due to sepsis [2]. Sites commonly reported include the abdomen, buttocks, lower extremities, and digits of susceptible individuals. Penile calciphylaxis continues to be a rare finding in medical literature with a reported incidence rate of 6% and is considered to be a poor prognostic indicator of disease progression [3].

Management is a shared decision between the patient and physician, taking into account the clinical presentation, co-morbidities, and wishes of the patient. Medical management includes addressing both hypercalcemia and hyperphosphatemia with the use of non-calcium/non-aluminum containing phosphate binders, hyperbaric oxygen therapy to promote wound healing, and the antioxidant cation chelator sodium thiosulphate which has been shown to increase the solubility of calcium deposits [4]. Surgical management includes total/partial penectomy or wound debridement [5]. 

We report a case of penile calciphylaxis in a 58-year-old male with a past medical history significant for end-stage renal disease on hemodialysis, diabetes mellitus, and hypertension. The patient presented with a painful lesion on the glans penis which rapidly progressed to gangrene with wet features. Following successful penile debridement he was discharged from the hospital with instructions to follow up with his urological surgeon on an outpatient basis for continued monitoring or distal penectomy. The patient has given written informed consent relating to his case to be reported in a medical publication.

## Case presentation

A 58-year old man presented to the emergency department with a three-week history of scrotal edema and a progressively painful penile lesion as well as a two-week history of daily watery diarrhea which has been intermittently black in color for the past two days, prompting his emergency department visit. He denied any nausea, vomiting, fevers, abdominal pain, recent travel, dysuria, hematuria, penile discharge, history of sexually transmitted infections, recent antibiotics, new medications, new food, history of gastrointestinal bleeds and liver disease. The patient denied current intravenous drug, alcohol or tobacco use. He had a history of end-stage renal disease currently being treated with hemodialysis three times per week for the past six-months, diabetes mellitus, hypertension, congestive obstructive pulmonary disease on 1 liter home oxygen and heart failure with reduced ejection fraction. He endorsed adherence to a renal diet with consistent carbohydrates and compliance with his home medications which include aspirin 81 mg once daily, bumetanide 2 mg once daily, clonidine 0.2 mg four times a day, insulin lispro 100 unit/ml 0-12 units subcutaneous injection with meals, semglee 100 units/ml 12 unit subcutaneous injection every night at bedtime, and sevelamer carbonate 800 mg three times a day. His vital signs showed a skin temperature of 36.8 degrees celsius, respiratory rate of 18 cycles per minute, oxygen saturation of 95% on room air, heart rate of 89 beats per minute, and blood pressure of 167/89 mmHg. Physical exam showed a circumcised penis with a dry and calcified glans, erythematous swollen left scrotum which was exquisitely tender to light palpation, both the penis and scrotum demonstrated no moisture or purulence at the time of exam. 

The patient laboratory diagnostics showed evidence of microcytic anemia, leukocytosis, and elevated neutrophil-lymphocyte ratio. His metabolic panel showed an anion gap metabolic acidosis and worsening kidney function. Table 1 summarizes the patient’s labs. Stool polymerase chain reaction (PCR) detected the rotavirus antigen. The patient’s diarrhea secondary to a rotavirus infection was treated with intravenous fluids.

**Table 1:** Serum laboratory results on the first and last days of admission.

Parameter	Reference range and units	Day 1 of admission	Last day of admission
White blood cell count (WBC)	4.0 - 10.0 10^3/uL	11.3	12.5
Red blood cell count (RBC)	4.3 - 5.9 10^6/uL	3.56	3.07
Hemoglobin	14.0 - 18.0 g/dL	10	8.5
Hematocrit	39 - 49%	31	27.4
Mean corpuscular volume	80.0 - 99.0 fL	87	89.2
Red cell distribution width	11.4 - 14.6%	18.5	18.2
Platelet count	150 - 400 10^3/uL	209	208
Lymphocytes	16.0 - 45.0%	5	6
Neutrophils relative percent	42.0 - 75.0%	85	85
Monocytes	2.0 - 12.0%	10	8
Eosinophils	0.0 - 0.5%	1	1
Basophils	0.0 - 2.0%	1	0
Sodium	135 - 145 mmol/L	136	137
Potassium	3.5 - 5.1 mmol/L	5	4.6
Cholride	98 - 107 mmol/L	94	99
Carbon dioxide	21 - 32 mmol/L	18	25
Glucose	74 - 106 mg/dL	129	133
Blood urean nitrogen (BUN)	7.0 - 18 mg/dL	112.3	45.8
Creatinine	0.70 - 1.30 mg/dL	7.6	4.8
Calcium	8.5 - 10.1 mg/dL	7.5	7.8
Aspartate aminotransferase (AST)	15 - 37 U/L	32	15
Alanine transaminase (ALT)	16 - 61 U/L	31	9
Protein, total	6.4 - 8.2 gm/dL	8	7
Albumin	3.4 - 5 gm/dL	3.1	2.5
Alkaline phosphatase	40 - 150 U/L	751	774
Bilirubin, total	0.3 - 1.00 mg/dL	0.8	0.6
International normalized ratio	1	1.2	1.2
Prothrombin (PT)	9.4 - 12.5 seconds	13.7	13.7
Troponin	<0.033 ng/mL	0.145	0.135
Lactic acid	0.5 - 2mmol/L	2.2	1.1

**Figure 1:**
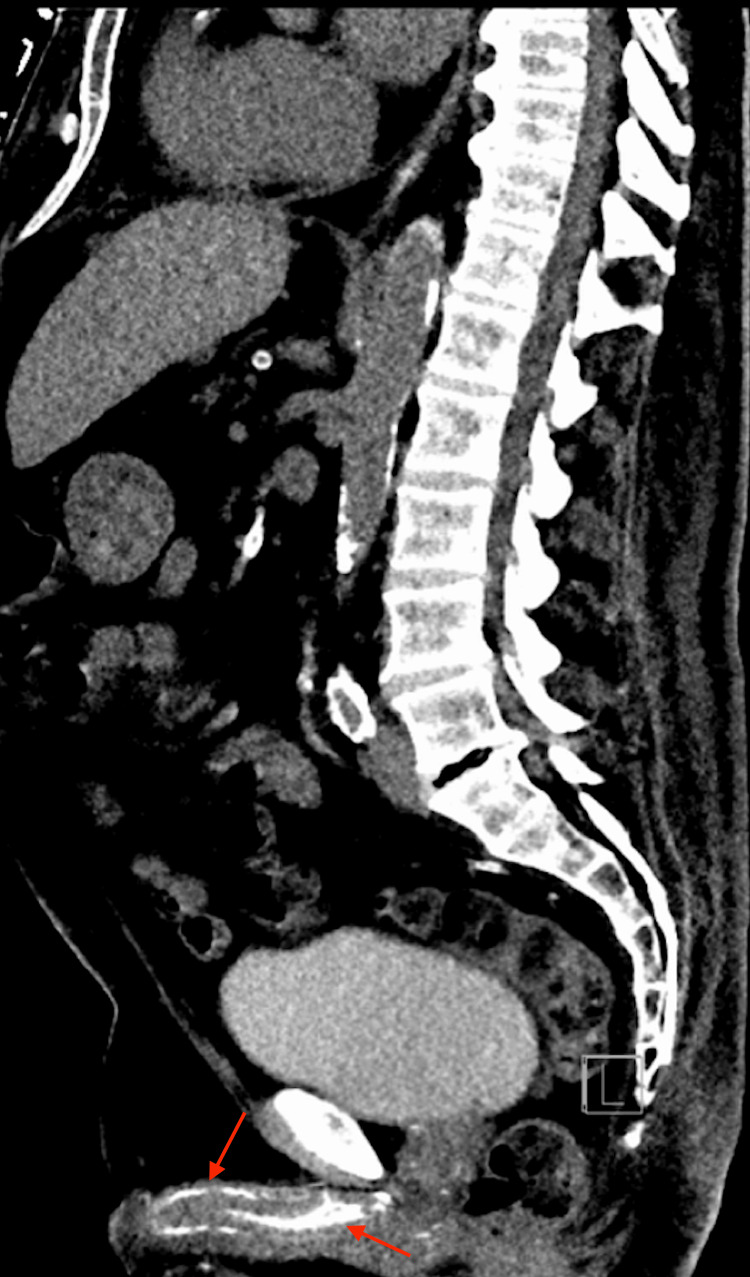
CT Abdomen and Pelvis without contrast, sagittal view demonstrating calcification within the penile vasculature.

The differential diagnosis for his penile lesion included but was not limited to cellulitis, penile calciphylaxis, and necrotizing infection. The patient was started on doxycycline 100 mg twice per day and ceftriaxone 1000 mg once daily. Laboratory risk indicator for necrotizing fasciitis (LRINEC) Score was 4 (positive for hemoglobin <11 and creatinine > 1.6), indicating low risk for necrotizing soft tissue infection. Ultrasound scrotum was negative for epididymitis, hydrocele, or varicocele. It was only positive for scrotal wall edema. The patient was noted to have a computed tomography (CT) abdomen and pelvis without intravenous contrast 3 months prior that demonstrated diffuse calcifications within the penile vasculature (Figure 1), aorta and major sub-branches. Based on clinical (diabetes and long-term dialysis), laboratory and radiographic findings, the diagnosis of penile calciphylaxis with dry gangrene of the distal glans was made. 

**Figure 2:**
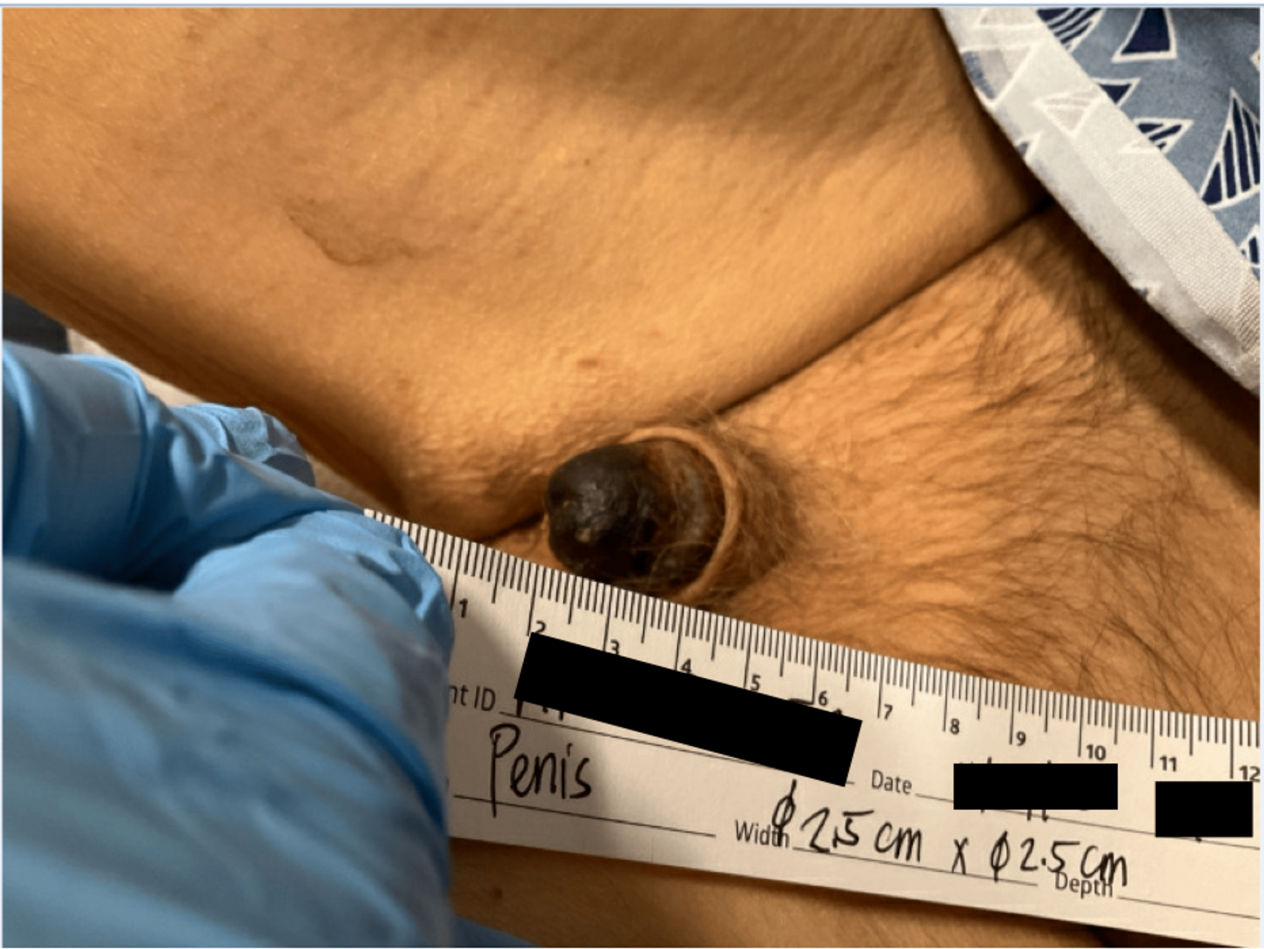
Postoperative penile debridement of gangrene with wet features.

The urology service was consulted for surgical intervention. The patient was educated on treatment options, which included conservative management with local wound care and antibiotics versus partial penectomy. The patient expressed a strong desire for conservative management. Despite being on broad-spectrum antibiotics, the patient’s gangrene of the distal penile glans continued to progress to include wet features. The patient, again, denied distal penectomy and therefore, he was taken to the operating room for penile debridement without distal penectomy (Figure 2).

The intraoperative findings showed a layer of necrotic and sloughing tissue over the entire glans of penis. The necrotic tissue was removed using a metzenbaum scissor followed by a 15 blade scalpel to scrape off any remaining necrotic tissue until viable bleeding tissue was encountered. A foley catheter was inserted and sterile dressing was applied. The patient was extubated in the operating room and taken down to the medicine floor for dressing changes with wound care. The urology service continued to monitor for acute changes in laboratory values and daily physical exams reflecting infection or ischemia in case further debridement is needed or if the patient was willing to proceed with penectomy. No acute changes had developed necessitating urologic intervention during the patient's hospital stay. Total length of inpatient hospital stay was approximately 3 days. He was encouraged to follow up with his urological surgeon in the outpatient setting for a postoperative visit or return to the hospital if he would like to pursue the recommended distal penectomy. He was also started on sodium thiosulfate 25 g intravenously three times per week after hemodialysis for his calciphylaxis. The patient followed up with urology 1 week after discharge, physical examination of the surgical site demonstrated adequate tissue perfusion and wound granulation devoid of necrotic tissue. During this visit the patient reaffirmed his decision of refusing distal penectomy for definitive therapy and desired to continue medical management with sodium thiosulfate 25 g intravenously three times per week.

## Discussion

Calciphylaxis, a rare and severe condition, involves the calcification and occlusion of microvasculature, leading to tissue ischemia and necrosis. It’s estimated that 1-4% of patients affected have end-stage kidney disease [6]. Penile calciphylaxis continued to be a rare case reported in medical literature and treatment continues to vary based on patient presentation. The treatment options for penile calciphylaxis include sodium thiosulphate, hyperbaric oxygen therapy, penectomy, vascular bypass, and parathyroidectomy [7]. A literature review showed that there is no association between sodium thiosulfate use, partial or total penectomy, and improvement in clinical outcomes [8]. At this time, physicians can attempt to combine different treatment modalities utilizing a multidisciplinary approach involving nephrology, urology, dermatology, wound care, plastic surgery, and pain management. 

The pathogenesis of calciphylaxis continues to be poorly understood with a number of proposed risk factors as well as theories [9]. Hyperparathyroidism secondary to vitamin D deficiency has been proposed to be a risk factor, as the reduced level of Vitamin D leads to a reduction in calcium absorption which in turn causes a rise in parathyroid hormone, ultimately raising levels of both phosphate and calcium which in theory promotes vascular calcifications. 

Histologically, calciphylaxis results in medial calcification, thrombosis, and obliteration of small arterioles within adipose tissues and dermal layers. Endothelial injury and the formation of microthrombi further propagate blood vessel occlusion, ischemia, necrosis, and ulceration [10]. Common sites include the abdomen, buttocks, and lower extremities. Penile calciphylaxis continues to be a rare finding with limited reports in medical literature and is considered to be a poor prognostic indicator of disease progression [2]. Medical versus surgical management in cases of penile calciphylaxis continues to be controversial. For the future, an area of study that would potentially reduce the prevalence of calciphylaxis and encourage timely management of penile calciphylaxis would include establishing risk factor criteria and the subsequent development of a scoring system to be utilized by physicians for at risk patients to promote disease awareness, prevent disease progression and obtain timely surgical or medical intervention. 

## Conclusions

Penile calciphylaxis continues to be a rare yet significant complication in chronic renal failure patients undergoing hemodialysis. Timely detection coupled with appropriate medical or surgical management can lead to favorable patient outcomes and a reduction in morbidity and mortality. Patients with known risk factors for systemic calciphylaxis should be educated on the caution of supplements known to induce hypercalcemic states, the benefits of a healthy lifestyle, and the warning signs of disease progression. As there are no current guidelines regarding definitive treatment, prevention and early intervention should be prioritized.
